# A poor prognostic male choriocarcinoma with multiple systemic metastases: a case report and the literature review

**DOI:** 10.3389/fmed.2024.1382672

**Published:** 2024-03-20

**Authors:** Wenpeng Huang, Zuohuan Zheng, Zheng Bao, Xiaoyan Xiao, Liming Li, Zhaonan Sun, Lei Kang

**Affiliations:** ^1^Department of Nuclear Medicine, Peking University First Hospital, Beijing, China; ^2^Department of Traditional Chinese Medicine, The Seventh People’s Hospital of Chongqing, Chongqing, China; ^3^Department of Radiology, The First Affiliated Hospital of Zhengzhou University, Zhengzhou, Henan Province, China; ^4^Department of Medical Imaging, Peking University First Hospital, Beijing, China

**Keywords:** male, primary choriocarcinoma, prognosis, computed tomography, ^18^F-FDG, PET/CT

## Abstract

**Background:**

Non-gestational choriocarcinoma, also known as primary choriocarcinoma, is extremely rare in men, manifesting with specific signs such as breast feminization, testicular atrophy, and loss of libido. The presentation typically includes elevated serum β-hCG levels, widespread metastatic disease, and a rapid progression of the condition.

**Case report:**

We present a rare case of a 41-year-old man diagnosed with choriocarcinoma, exhibiting a unique combination of multiple metastases, including lung, brain, bone, and retroperitoneal lymph node metastases, as confirmed by ^18^F-FDG PET/CT imaging. The patient was treated with aggressive chemotherapy and pembrolizumab, and the prognosis remained poor. The patient’s overall survival was a mere 5 months following diagnosis.

**Conclusion:**

Non-gestational choriocarcinoma represents a rare entity in clinical practice and should be considered in young men presenting with gynaecomastia and elevated β-hCG levels alongside normal gonads. Thus, we advocate for a more comprehensive inquiry into medical history and a systematic examination. The ^18^F-FDG PET/CT examination not only visually delineates the lesion’s location and extent but also serves as a cornerstone for clinical tumor staging, providing valuable support for treatment monitoring and subsequent follow-up.

## Introduction

Choriocarcinoma, a rare trophoblastic tumor, exhibits high invasiveness and is characterized by the proliferation and interstitial transformation of abnormal chorionic trophoblast cells. It is distinguished by the absence of a chorionic structure, accompanied by hemorrhage and necrosis, and has the capability to secrete beta-human chorionic gonadotropin (β-hCG). The condition can be classified into two main categories: gestational choriocarcinoma and non-gestational choriocarcinoma (primary choriocarcinoma) (1–5). Gestational choriocarcinoma originates from the trophoblast of various gestational events, such as hydatidiform mole, spontaneous abortion, and normal pregnancy. On the other hand, non-gestational choriocarcinoma, also known as primary choriocarcinoma, is extremely rare in men, manifesting with specific signs such as breast feminization, testicular atrophy, and loss of libido (2, 6, 7). This type of choriocarcinoma can be further categorized into gonadal choriocarcinoma (testis) and extragonadal choriocarcinoma (such as mediastinum and retroperitoneum) based on its origin and primary site (8, 9). The presentation typically includes elevated serum β-hCG levels, widespread metastatic disease, and a rapid progression of the condition (10, 11).

In this report, we present a rare case of a 41-year-old man diagnosed with choriocarcinoma, exhibiting a unique combination of multiple metastases, including lung, brain, bone, and retroperitoneal lymph node metastases, as confirmed by 2-Deoxy-2-[fluorine-18]-fluoro-D-glucose (^18^F-FDG) positron emission tomography combined with computed tomography (PET/CT) imaging. Through an extensive literature search on the PubMed database, covering the period from 1996 to 2023 and utilizing keywords related to choriocarcinoma and CT, the search was carried out with and without the addition of filters, such as English language only, type of article, and subjects, excluding duplicate papers. We identified a total of 53 relevant publications. The summarized findings are presented in Table 1 (12–64). Previous studies have overwhelmingly reported cases of choriocarcinoma in pregnant females. The current case of primary choriocarcinoma occurring in a male is exceptionally rare.

**Table 1:** ^18^F-FDG PET/CT and CT manifestations of primary choriocarcinoma.

Case	Authors	Gender	Age	Obstetric history	Medical history	Clinical symptoms	Max β-hCG, mIU/mL	Primary sites	Invasion and metastasis	Max diameter/cm	SUVmax	Management	Outcome
1	Lowe et al. (12)	M	18	None	Microcytic anemia	Black stools, lethargy, and dizziness	32,219	Testicles	Stomach, brain and kidney lungs	NA	NA	Surgery + chemotherapy	Alive
2	Maruoka et al. (13)	F	26 y	Molar pregnancy history at 20 y and spontaneous abortion history at 26 y.	NA	NA	233.8	Negative	Pulmonary metastasis	3.0	2.0	Surgery	Alive at 1 mo
3	Dose et al. (14)	F	37 y	Abortion at 34 y	Leiomyoma of the uterus, Cystic lesion of the right ovary	Irregular bleedings, and secondary sterility	8,000	Uterus	None	5	NA	Surgery + chemotherapy	Alive at 13 mo
4	Gazzilli et al. (15)	F	36 y	History of 4 spontaneous abortion	NA	Repeated epistaxis	3,839	Nasal and ethmoid	Sphenoidal and maxillary sinuses; cervical lymph node	NA	NA	Surgery + chemotherapy	Complete remission
5	Numnum et al. (16)	F	22 y	Molar pregnancy history at 16 and a term delivery at 19 y	GTD (gestational trophoblastic disease)	Vaginal bleeding and abdominal cramping	1,341	Left pelvis	None	NA	5.0	Surgery + chemotherapy	Alive at 8 mo
6	Hebart et al. (17)	F	32 y	NA	Irregular menstruation	Irregular periods, intermittent pleuritic chest pains and nonproductive cough	127,429	None	Left lung artery and right lung	NA	NA	Chemotherapy	Died within few hours
7	Hebart et al. (17)	F	29 y	Term delivery 4 mo ago.	NA	NA	400	None	Left and right lung	NA	NA	Surgery + chemotherapy	Alive at 1 y
8	Huang et al. (18)	F	40 y	Term delivery 10 y ago	Pseudoaneurysms, GTD	Headache	4,154	None	Left frontal lobe in brain and left lung	1.5	NA	Chemotherapy	Alive at 3 y
9	Tripathi et al. (19)	F	38 y	Term delivery 4 y ago	NA	NA	33,000	NA	Right atrium and lung	NA	NA	Chemotherapy + surgery	Alive after 5 cycles
10	Rao et al. (20)	F	28 y	Term delivery 4 y ago	NA	Irregular vaginal bleeding	1,004.69	Pancreas	None	5.4	3.9	Surgery + chemotherapy	Alive at 2 y
11	Huang et al. (21)	F	46 y	NA	NA	Right upper abdominal pain	59,283	Pancreas	Liver and inferior vena cava lymph nodes	7.4	17.7	Surgery +125I-seed implantation	Died at 10 mo
12	Trübenbach et al. (22)	F	33 y	Abortion 10 months ago	Pulmonary embolism, pleural effusion and pulmonary hypertension	Recurrent episodes of exertional dyspnoea and pleuritic chest pain	129,500	None	Left pulmonary artery	NA	NA	Methotrexate	Died at 1 d
13	Zhang et al. (23)	F	29 y	NA	Recurrent right-sided pneumothoraces	Recurrent right-sided pneumothoraces and episodic menorrhagia	NA	Left lung	NA	NA	7.2	Surgery	NA
14	FCimarelli et al. (24)	M	23	None	GCT (germ cell tumors)	Gynecomastia and abdomen pain	425,000	Retroperitoneal	Liver, lung and lomboaortic lymph nodes	14	NA	Surgery + chemotherapy	NA
15	Joshua et al. (25)	F	31 y	Term delivery	Dult polycystic kidney disease and mild hypertension	Pulmonary vein bleeding	91,348	Uterus	Lung and liver	NA	NA	Chemotherapy	Alive at 8 mo
16	Sone et al. (26)	F	31 y	NA	NA	NA	NA	NA	Lung	NA	7.3	Chemotherapy	Died
17	Kidd et al. (27)	M	48 y	None	NA	Blurred vision and headache	NA	NA	Brain and lung and	NA	NA	Supportive therapy	Died at 10 d
18	Aleem et al. (28)	F	30 y	NA	NA	A lump in right breast and right axilla	NA	Uterus	Right breast	7	5.9	Surgery + chemotherapy	alive
19	Goldfarb et al. (29)	F	50 y	Gravida 3, para 2, abortus 1	NA	An intra-uterine mass and hemorrhaged during an office endometrial biopsy	28,725	Uterus	Lungs	NA	NA	Surgery + chemotherapy + targeted therapy	Disease progression at 2 y
20	Du et al. (30)	F	37 y	One term delivery and two miscarriages	Irregular menstruation	Hemoptysis	279,064	NA	Lung, renal and brain	5.8	18.3	Surgery + chemotherapy	Died at 1 y
21	Wang et al. (31)	F	21 y	Miscarriage at 20 y.	NA	Chest pain and fever	>10,000	NA	Lung	NA	27.5	Chemotherapy	Alive at 1 y
22	Sekine et al. (32)	M	49 y	None	Diabetes mellitus and hepatitis C	Abdominal pain and fever	5,3,000	Liver	Lung	NA	NA	None	Died at 60 d
23	Sironi et al. (33)	F	38 y	No obstetric history in the past 10 y	NA	NA	210	NA	Lung	NA	NA	Surgery + chemotherapy	Alive at 12 mo
24	Sironi et al. (33)	F	29 y	Recent molar pregnancy history	NA	NA	3,471	NA	Lung	0.5	Poor FDG avidity	Surgery	Alive at 6 mo
25	Sironi et al. (33)	F	49 y	Molar pregnancy 6 y ago	NA	NA	>10,000	NA	Left lung and liver	13	7.98	Surgery + chemotherapy	Alive
26	Gasparri et al. (34)	F	37 y	Term delivery 1 y ago	Enhancement of beta-human chorionic gonadotropin levels	Period suspension	NA	NA	Lung	1.1	NA	Surgery + chemotherapy	Alive at 2 y
27	Gvinianidze et al. (35)	F	42 y	Term delivery 6 mo ago	NA	Cough, shortness of breath on physical exertion and haemoptysis	3,200	NA	Lung and brain	NA	NA	Surgery + chemotherapy	Alive at 8 mo
28	Snoj et al. (36)	F	35	Three spontaneous abortions in the past year	NA	Right chest pain	169,396	Lung	Brain	6.4	2.7	Surgery + chemotherapy + radiotherapy	Alive at 1 y
29	Göksel et al. (37)	M	27	None	Anemia and melena	Anemia and melena	NA	Testicles	Stomach	NA	NA	NA	NA
30	Francischetti et al. (38)	M	41	None	Transient ischemic attack and stroke	Recurrent numbness of right finger and arm, left facial weakness and language difficulty	12,118	Mediastinum	Brain	9	NA	NA	NA
31	Pakkala et al. (39)	F	23	At 28 weeks of gestation	NA	Right hypochondrium pain	11,875	NA	Liver and pulmonary, presacral, rectal, and iliac nodal metastasis	11.6	NA	Termination of pregnancy + surgery + chemotherapy	Alive at 7 mo
32	Su et al. (40)	F	31	NA	Right ovarian dermoid cyst and bilateral polycystic ovaries	NA	790,000	Uterus	Lung	0.4	Poor FDG avidity	Surgery + chemotherapy	NA
33	Lee et al. (41)	F	15	None	NA	Uterine bleeding	76,600	Right ovary	None	8.5	NA	Surgery + chemotherapy	Alive at 6 y
34	Lehmann et al. (42)	F	32	Term delivery 5 mo ago	Gestational choriocarcinoma	Uterine bleeding	13,575	Uterus	Lung and brain	NA	NA	Surgery + chemotherapy	Alive at 2 y
35	Usta et al. (43)	F	28	Term delivery 4 y ago	NA	Lower pelvic pain, flank pain, vaginal bleeding and a delayed menstrual period	70,373	Renal artery	NA	NA	NA	Surgery + chemotherapy	Alive at 1 y
36	Clair et al. (44)	F	30	A 15-week twin pregnancy consisting of a normal fetus and a suspected molar gestation	NA	NA	96,952	Uterus	Lungs	3.2	NA	Surgery + chemotherapy + radiotherapy + targeted therapy	Alive at 5 y
37	Dance et al. (45)	M	37 d	None	Anemia and thrombocytopenia	A pedunculated, friable red glabellar mass	NA	NA	Cutaneous, liver, and lung	6.8	10.3	Surgery + liver transplant + chemotherapy	Alive at 3 mo
38	Shaw et al. (46)	F	38 y	An abortion 11 mo ago	Pulmonary tuberculosis	Oliguria and leg edema	382	Uterus	Stomach	NA	3.63	Surgery	Alive at 15 mo
39	Hyun et al. (47)	F	38	Molar pregnancy history 4 y ago	Hydatidiform mole and asymptomatic recurrent pneumothorax	NA	NA	Uterus	Lung	NA	Poor FDG avidity	Surgery + chemotherapy	Alive at 22 mo
40	Pessanha et al. (48)	M	14	None	Tyrosinemia type I, HCC, Budd-Chiari Syndrome with partial occlusion of the hepatic veins and micronodular hepatic cirrhosis	Weight loss, minor edema of the lower limbs, moderate gynecomastia, and morning nausea	1,984	Liver	Lung	6.7	Poor FDG avidity	Surgery + chemotherapy	Alive at 11 y
41	Chen et al. (49)	F	43	Term delivery 3 y ago	Ectopic pregnancy	Low abdomen pain	5,241	NA	Liver, lungs, marrow cavity, thoracic vertebra and brain	5.8	6.4	Surgery + chemotherapy	Alive at 32 mo
42	Kohler et al. (50)	M	64 y	None	NA	Abdomen pain and intra-abdominal bleeding	>5,000	Liver	Lungs and iliacal lymph nodes	14.5	NA	Surgery + chemotherapy	Died at 6 mo
43	Dlewati et al. (51)	F	54	NA	Hypertension and ischemic stroke	Dyspnea, cough, and intermittent hemoptysis	98,138	Lung	Right breast and brain	4.8	3.1	Surgery + chemotherapy + radiotherapy	NA
44	Dasgupta et al. (52)	M	29	None	NA	A lump on the neck	18	Testicle	Cervical lymph node	4.8	NA	Surgery	NA
45	Huang et al. (53)	F	32	Molar pregnancy 7 mo ago	Hydatidiform mole and GTD	NA	17,094	Uterus	Anterior pelvic peritoneal wall	NA	4.7	Surgery + chemotherapy	Alive at 30 mo
46	Chatterjee et al. (54)	F	41	Molar pregnancy history	NA	Headache and left eye pain	7,062	NA	Endocranium	3.5	Poor FDG avidity	Surgery + chemotherapy	Alive at 3 y
47	Zhou et al. (55)	F	66	NA	NA	Abdominal discomfort	795.14	Stomach	Liver	2.0	NA	Surgery + chemotherapy + radiotherapy	Died at 8 mo
48	Matsuo et al. (56)	F	46	Term delivery 5 y ago	NA	Hemoptysis	132	NA	Lung	4	NA	Surgery + chemotherapy	Alive at 8 mo
49	Røge et al. (57)	M	71	None	Concurrent goserelin-treated metastasized prostate adenocarcinoma	Back pain	NA	Mediastinum	Lungs	5.5	NA	Surgery	Died
50	Kazemi et al. (58)	F	49	Gravida 2, para 2	Asthma, pulmonary edema, and hyperlipidemia	Cough and right upper quadrant pain	79,000	NA	Liver and lungs	NA	NA	Chemotherapy + target therapy	Died
51	Pan et al. (59)	M	19	None	NA	Chest pain and occasional cough	>200,000	Mediastinum	Lungs, brain, liver, spleen, kidney, and skeletal	8.8	NA	Surgery + chemotherapy	Died at 4 mo
52	Kamata et al. (60)	M	70	None	NA	Cough	NA	Lung	NA	3.8	6.5	Surgery	Alive at 2 y
53	Iijima et al. (61)	F	45	Molar pregnancy history 6 y ago	Hydatidiform mole	Irregular vaginal bleeding	482.8	Uterus	Lung	NA	9.57	Surgery + chemotherapy	Alive at 7 y
54	Horotsu et al. (62)	M	78	None	NA	NA	120	Stomach	Liver	3	5.4	Surgery + chemotherapy	Died at 10 mo
55	Rehman et al. (63)	M	50	None	NA	Painful bilateral gynaecomastia	3,756	Mediastinum	Lung	NA	NA	Surgery + chemotherapy	Alive at 3 y
56	Guo et al. (64)	F	61	Term delivery at 31 y	NA	Vaginal bleeding	9273.9	Vagina	Lung	3	Poor FDG avidity	Surgery + chemotherapy	Alive at 4 y

## Case presentation

A 41-year-old man presented with diminished appetite symptoms a year ago, but he did not undergo an examination. His condition deteriorated 2 months ago, marked by malaise, night sweats, and lower back pain, leading to a weight loss of 10.5 kg over 1 year. The patient was hospitalized for a physical examination, revealing bilateral breast development and a palpable mass measuring approximately 12 cm × 10 cm with poor mobility in the right abdomen. Laboratory tests indicated a white blood cell count of 12.00 × 109/L (normal range 3.5–9.5 × 109/L), D-dimer of 5.94 mg/L (normal range 0–0.5 mg/L), fibrinogen of 5.10 g/L (normal range 2–4 g/L), C-reactive protein of 99.90 mg/L (normal range 0–10 mg/L), and CA-reactive protein of 99.90 mg/L (normal range 0–10 mg/L). Additionally, CA-199 was elevated at 47.76 U/mL (normal range 0–37 U/mL), non-small cell lung cancer antigen 21–1 was elevated at 28.16 ng/mL (normal range 0–3 ng/mL), neuron-specific enolase was elevated at 67.66 ng/mL (normal range 0–16.3 ng/mL), prolactin was elevated at 35.62 ng/mL (normal range 2.1–17.7 ng/mL), estradiol was lower than normal at 26.33 pg./mL (normal range 35–245 pg./mL), testosterone was elevated at 179.32 ng/mL (normal range 1.75–7.81 ng/mL), beta-human chorionic gonadotropin (β-hCG) was markedly elevated at 937,268.00 mIU/mL (normal range 0–5 mIU/mL), and EBV antibody IgG was 4.06 (positively expressed).

**Figure 1:**
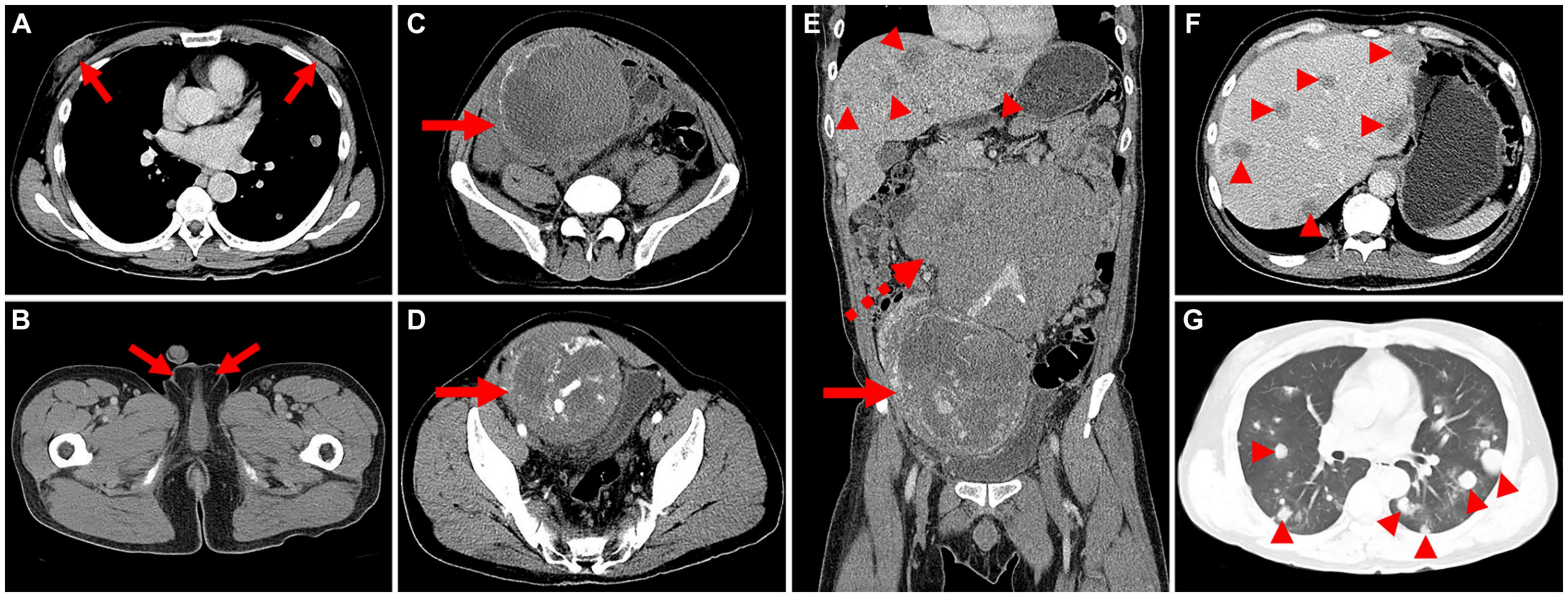
Computed tomography (CT) images of male choriocarcinoma with multiple systemic metastases (December 15, 2018). **(A)** The transverse image reveals bilaterally enlarged breasts (long arrows). **(B)** The transverse image displays empty scrotums bilaterally, with the testes not visualized (long arrows). **(C)** A roundish mass with heterogeneous density is observed in the pelvis, featuring eggshell-like calcifications at its margins (long arrows). **(D)** The transverse image in the arterial phase shows heterogeneous mild enhancement of the lesion, with evident thickening of blood-supplying arteries, areas of necrotic cystic degeneration in the interior, and tortuous, dilated veins in the surrounding area (long arrows). **(E)** The coronal image in the venous phase reveals multiple enlarged lymph nodes in the retroperitoneum, merging with one another (dashed arrows). **(F)** The transverse image in the venous phase exhibits multiple hypodense foci in the liver, characterized by ring-shaped and mild enhancement (arrowheads). **(G)** The transverse image depicts multiple rounded hyperdense nodules in both lungs (arrowheads).

The patient underwent an enhanced CT examination, revealing bilateral enlarged breasts (Figure 1A), empty scrotums bilaterally, with the testes not visualized (Figure 1B). A roundish mass with heterogeneous density was observed in the pelvis, post-enhancement, the mass exhibited heterogeneous mild enhancement (Figures 1C,D). Multiple enlarged lymph nodes were identified in the retroperitoneum, merging with one another, encircling the abdominal aorta and vessels, resulting in displacement of surrounding organs (Figure 1E). Additionally, multiple hypodense foci, characterized by ring-shaped and mild enhancement, were detected in the liver (Figure 1F). Furthermore, multiple rounded hyperdense nodules were observed in both lungs (Figure 1G). Following a thorough physical examination, laboratory tests, and CT imaging, the clinician initially suspected the presence of seminomas and metastases.

**Figure 2:**
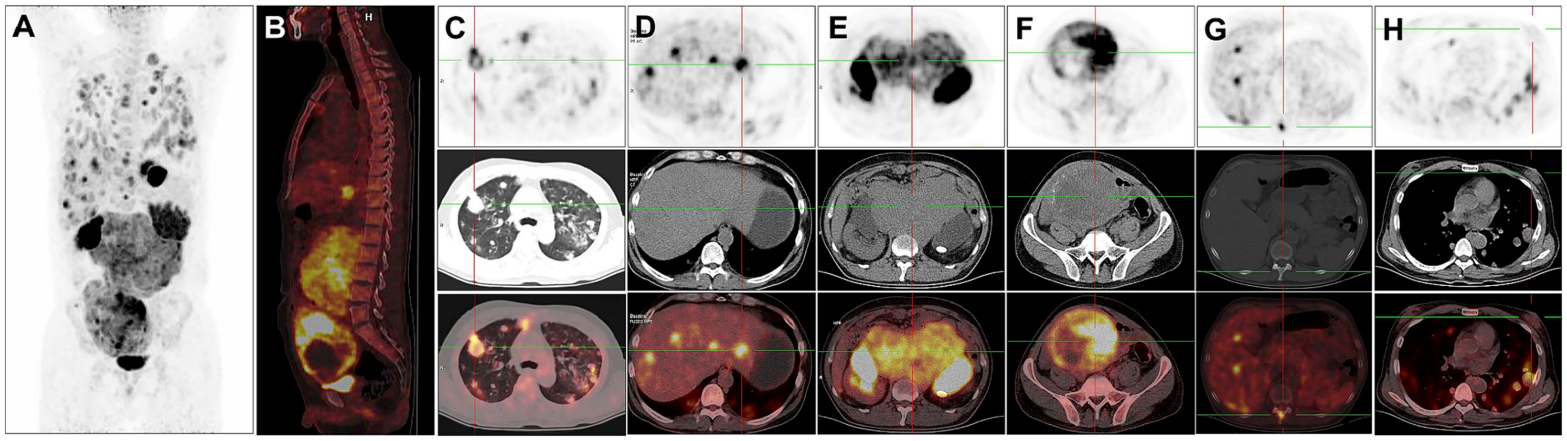
^18^F-FDG PET/CT images of male choriocarcinoma with multiple systemic metastases (December 20, 2018). **(A)** The anteroposterior 3-dimensional maximum intensity projection (MIP) image demonstrates increased metabolic activity in the both lungs, abdominal cavity, and pelvic cavity. **(B)** The sagittal fusion image demonstrates increased metabolic activity in the liver, retroperitoneum and pelvis. **(C)** Transverse images reveal multiple soft tissue nodules in both lungs with significantly increased ^18^F-FDG uptake (SUVmax = 12.7). **(D)** Transverse images reveal the liver exhibiting multiple slightly hypodense nodules and masses, characterized by markedly increased ^18^F-FDG uptake (SUVmax = 38.6). **(E)** Transverse images depict, in the retroperitoneum, numerous intermingled soft tissue masses with markedly increased ^18^F-FDG uptake (SUVmax = 13.1). **(F)** Transverse images identify a soft tissue mass of irregular shape in the right pelvis, displaying unevenly increased ^18^F-FDG uptake (SUVmax = 19.5). The maximum dimensions of this mass are approximately 12.0 cm × 15.2 cm, with areas of cystic necrosis and calcifications noted. **(G)** Transverse images reveal markedly increased ^18^F-FDG uptake at the thoracic 11 vertebral attachments (SUVmax = 11.8). **(H)** Transverse images show the bilateral breast glands exhibiting thickening with a slight increase in ^18^F-FDG uptake (SUVmax = 1.2).

For staging, the patient underwent further ^18^F-FDG PET/CT (Figures 2A,B). The ^18^F-FDG PET/CT scan, performed utilizing Siemens Biograph Truepoint 64 PET/CT machine, was conducted 60 min after the intravenous administration of ^18^F-FDG (6.1 mCi), revealing multiple soft tissue nodules in both lungs with significantly increased ^18^F-FDG uptake (SUVmax = 12.7, Figure 2C). The liver exhibited multiple slightly hypodense nodules and masses, characterized by markedly increased ^18^F-FDG uptake (SUVmax = 38.6). The larger liver mass measured approximately 3.6 cm × 2.1 cm (Figure 2D). In the retroperitoneum, numerous intermingled soft tissue masses with markedly increased ^18^F-FDG uptake (SUVmax = 13.1) were observed, with the largest dimension measuring about 16.2 cm × 16.3 cm. Additionally, patchy calcifications were evident within this area (Figure 2E). A soft tissue mass of irregular shape was identified in the right pelvis, displaying unevenly increased ^18^F-FDG uptake (SUVmax = 19.5). The maximum dimensions of this mass were approximately 12.0 cm × 15.2 cm, with areas of cystic necrosis and calcifications noted (Figure 2F). Markedly increased ^18^F-FDG uptake (SUVmax = 11.8) was detected at the thoracic 11 vertebral attachments (Figure 2G). Notably, no testes were visualized in the bilateral scrotum. The bilateral breast glands exhibited thickening with a slight increase in ^18^F-FDG uptake (SUVmax = 1.2, Figure 2H).

**Figure 3:**
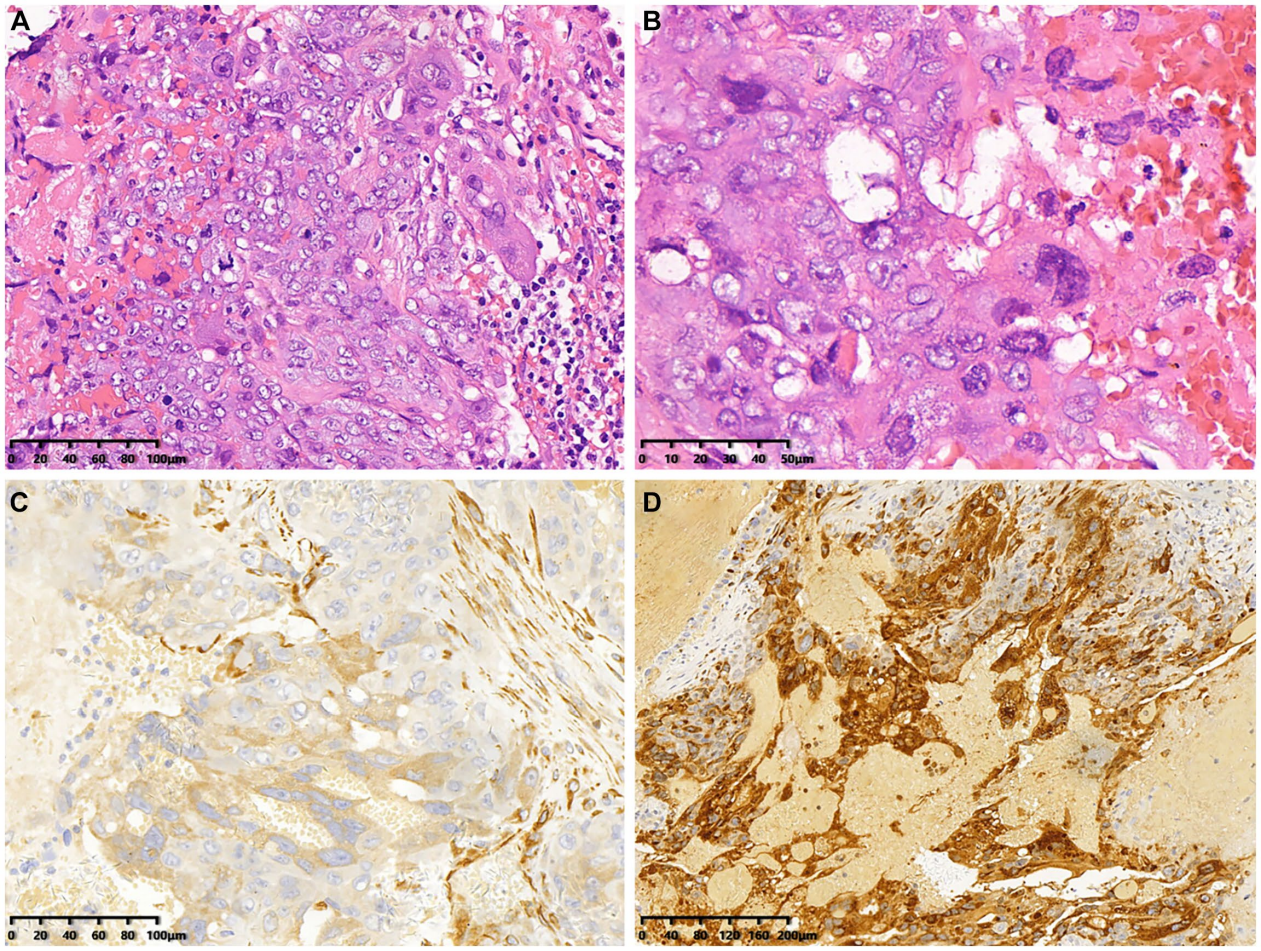
Histopathological and immunohistochemical images of choriocarcinoma (December 22, 2018). **(A,B)** Hematoxylin–eosin (HE) staining (magnification ×200 and 400) showed extensive hemorrhage and characteristic features indicative of choriocarcinoma. **(C,D)** Immunohistochemistry showed that the tumor cells were positive for HPL and β-HCG (magnification ×200).

The patient underwent CT-guided puncture biopsy of a lesion on the liver, extensive hemorrhage was seen microscopically with typical features of choriocarcinoma. The tumor consists of mononuclear cytotrophoblasts and multinucleated syncytiotrophoblasts (Figures 3A,B). Immunohistochemistry showed positive expression of HPL (Figure 3C), β-HCG (Figure 3D), CD34 (vascular), CK7, CK19 and SALL-4. In addition, Ki-67 was observed to be positive in 90% of the tumor cells. The pathologic diagnosis confirmed choriocarcinoma metastasis.

**Figure 4:**
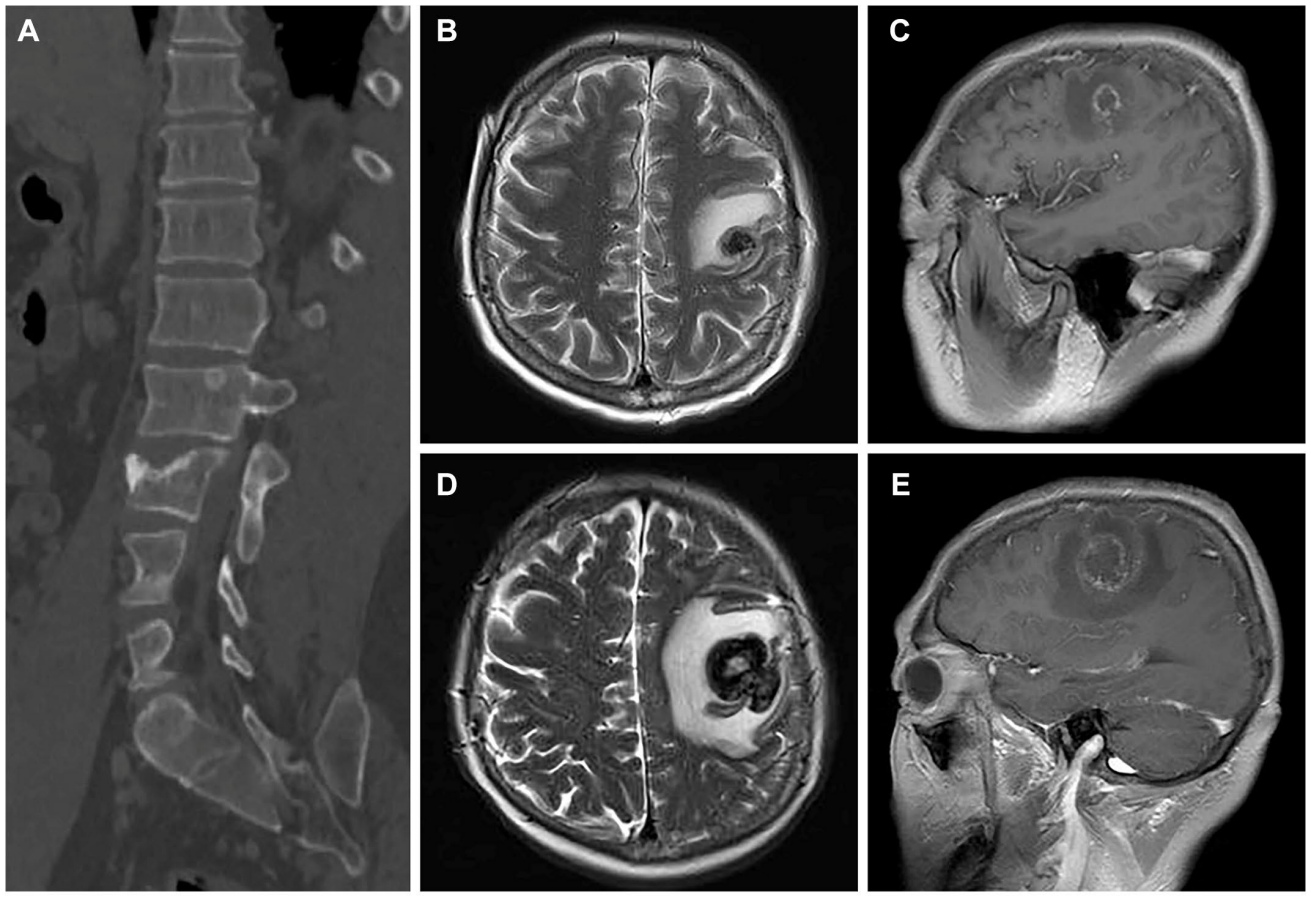
CT images and magnetic resonance images after treatment for choriocarcinoma (May 5 & 14, 2020). **(A)** The sagittal bone window CT image reveals multiple metastases in the vertebral body. **(B,C)** The T2-weighted imaging (T2WI) and enhanced images indicate the development of brain metastases in the patient. **(D,E)** Subsequent scans after 9 days demonstrate an enlargement and increased size of the patient’s brain metastatic lesions compared to the previous images in both T2WI and enhanced sequences.

After confirming the absence of contraindications to chemotherapy, the patient underwent treatment with an eight-cycle regimen consisting of etoposide (200 mg/d1–4), cisplatin (40 mg/d1–3), and bleomycin (30 U/d1,5). According to RECIST guidelines, subsequent CT examinations indicated stable disease (SD) in the patient’s status. Considering the patient’s chemotherapy tolerance, a collaborative decision was made to initiate a four-cycle course of anti-tumor therapy using the PD-1 antibody (pembrolizumab). Unfortunately, the patient experienced adverse effects of diarrhoea during this treatment phase. At the conclusion of the Avelumab treatment, follow-up CT scans revealed an increase in size of metastatic lesions in both lungs and liver, accompanied by the emergence of multiple metastases in the vertebral body (Figure 4A). Due to an inadequate response to pembrolizumab, the patient was subsequently treated with the regimen of “methotrexate (1.5 g) + actinomycin (0.4 mg).” Two weeks post-treatment initiation, the patient presented with dizziness and headache. A cranial MRI disclosed a rounded lesion in the left frontal lobe with high signal on T2WI (Figure 4B), measuring approximately 1.7 cm in diameter, and exhibiting inhomogeneous annular enhancement (Figure 4C). Nine days later, the patient’s secondary epileptic symptoms exacerbated, with a repeat MRI showing an enlarged frontal lobe lesion surpassing its previous size (Figure 4D). Additionally, new bilateral occipital lobe metastases were evident (Figure 4E). Despite medical recommendations, the patient declined further treatment and succumbed to the illness 2 weeks later. The patient’s overall survival was a mere 5 months following diagnosis.

## Discussion

Germ cell tumors encompass various cell types, broadly categorized into seminoma and non-seminoma. Non-seminomatous germ cell tumors (NSGCTs) exhibit four subtypes: embryonal carcinoma, yolk sac tumor, teratoma, and choriocarcinoma, often displaying a combination of seminomatous and non-seminomatous components. Choriocarcinoma, the rarest subtype, lacks a clear etiology in males, potentially associated with various risk factors such as Klinefelter syndrome, cryptorchidism, exposure to radiation, and more. Cryptorchidism may be one of the significant factors contributing to testicular choriocarcinoma. It has been reported that the likelihood of choriocarcinoma in cryptorchidism patients is 20–40 times higher compared to individuals with normal testes (65, 66). The absence of the testicle in this particular case may be a significant factor contributing to the development of testicular choriocarcinoma.

Due to its pronounced invasiveness into blood vessels and tissues, extensive hemorrhage, necrosis, and lymphovascular invasion are common findings (67). Choriocarcinoma primarily metastasizes hematogenously, resulting in early and extensive dissemination to sites such as the lungs, liver, skin, retroperitoneal lymph nodes, gastrointestinal tract, and central nervous system (37, 68–71). In this case, the diagnosis of choriocarcinoma was established at an advanced stage, with multiple metastases already present throughout the body. A thorough medical history and examination are necessary to detect the primary lesion. Characteristics of the typical choriocarcinoma patient history include pregnancy-relatedness, elevated β-hCG levels, abnormal uterine bleeding, and vaginal bleeding. Patients with choriocarcinoma usually have a history of multiple pregnancies. Primary choriocarcinoma is extremely rare in men, manifesting with specific signs such as breast feminization, testicular atrophy, and loss of libido.

The determination of serum tumor markers, specifically serum β-hCG and AFP, proves beneficial in choriocarcinoma diagnosis as they are elevated in approximately 80% of cases. Our case report demonstrates markedly elevated serum β-hCG, produced by syncytiotrophoblasts, indicating its diagnostic relevance. Monitoring the serum concentration of β-hCG also aids in assessing treatment response. According to the International Cooperative Organization for Germ Cell Cancer, a β-hCG level exceeding 50,000 mIU/mL signifies a poor prognosis. Some patients may manifest hyperthyroidism or bilateral gynecomastia, attributed to markedly elevated serum β-hCG levels, often exceeding 50,000 mIU/mL (66, 72). In our case, elevated β-hCG levels stimulated supraphysiological testosterone secretion, subsequently aromatized to estradiol, resulting in gynecomastia. Following chemotherapy, a substantial decrease in β-hCG levels was observed, aligning with existing literature. In addition to the abnormal laboratory results, elevated white blood cell count may indicate an underlying infection or inflammation. Elevated levels of D-dimer, C-reactive protein, and fibrinogen may suggest a hypercoagulable state, which could be related to the patient’s malignancy. Elevated tumor markers and neuron-specific enolase levels may indicate an underlying malignancy. Elevated prolactin and testosterone levels and a low estradiol level may suggest an endocrine disorder, potentially related to the patient’s breast development and gynecomastia. Furthermore, positive EBV antibody IgG suggests a previous Epstein–Barr virus infection, which may have contributed to the patient’s condition.

The pathogenesis of extragonadal choriocarcinoma has been a subject of prolonged debate, currently revolving around three hypotheses (73). First, the tumor is postulated to arise from retained primordial germ cells that undergo abnormal migration during embryogenesis (74). Second, it is proposed that the lesion originates from the transformation of a nontrophoblastic neoplasm (75). The third hypothesis suggests that the tumor results from the metastasis of choriocarcinoma from the gonads, accompanied by the spontaneous regression of the primary choriocarcinoma in the gonads.

Histologically, choriocarcinoma is distinguished by a biphasic pattern featuring mononucleated cytotrophoblast cells (including intermediate trophoblast cells) alongside multinucleated syncytiotrophoblasts, with an absence of chorionic villi. The former cells organize lamellarily to form a villous structure, while the latter secrete β-hCG and human placental lactogen (HPL), observable at the tumor progression margin (76–78). Immunohistochemistry plays a crucial role in the differential diagnosis of relevant diseases (79). GATA binding protein 3 (GATA-3), Spalt-like transcription factor 4 (SALL-4), Cytokeratin (CK) AE1/AE3, and 3-beta-hydroxysteroid dehydrogenase type 1 (HSD3B1) have been identified as potential immunohistochemical markers for gestational choriocarcinoma (80, 81). A high Ki-67 proliferation index is noted in over 90% of choriocarcinoma cases (1). Immunohistochemistry holds significance in the differential diagnosis of relevant diseases.

The imaging characteristics of choriocarcinoma lack distinctive features that differentiate it from other types of germ cell tumors, making its initial diagnosis challenging. However, a comprehensive pre-operative workup, including clinical imaging assessments, remains crucial. There is a scarcity of studies reporting imaging features of retroperitoneal choriocarcinoma in men (82). On CT scans, the lesion typically exhibits a large central necrosis with common occurrences of bleeding and ring enhancement of solid components at the tumor margins. MRI findings include a mixed high signal on T1WI and T2WI associated with combined hemorrhage. Striped hypointensity on both T1WI and T2WI at the tumor margin suggests the possibility of old hemorrhage. In our presented case, CT revealed a cystic solid tumor with eggshell-like calcification at the margin, and an enhancement scan displayed circumferential enhancement, with patchy non-enhanced necrotic areas within the lesion. Due to the rapid growth of choriocarcinoma, histologically, the lesion lacks interstitial blood vessels, relying on the invasion of blood vessels for nutrition, leading to frequent internal necrosis. While CT served as the initial diagnostic modality in our case study, it proved insufficient in revealing metastatic details.

^18^F-FDG PET/CT emerges as a noninvasive tool, showcasing its exceptional utility in discerning the metabolic activity of tumors. It proves particularly advantageous in delineating the staging of choriocarcinoma, tracking relapse, and assessing therapeutic response (18, 24, 33, 47). PET/CT effectively identifies occult choriocarcinoma lesions that may elude detection through conventional imaging methods such as MRI or CT (30). In the case under scrutiny, ^18^F-FDG PET/CT provided comprehensive insights into the extent of systemic involvement of the tumor. Another pivotal role of ^18^F-FDG PET/CT in choriocarcinoma management lies in its ability to monitor treatment response. Radiological assessment using ^18^F-FDG PET/CT should be incorporated at the end of treatment and annually during follow-up. The existing literature on choriocarcinoma is predominantly comprised of case reports, highlighting its heightened metabolic state with significant ^18^F-FDG uptake, indicative of robust trophoblastic proliferation and the tumor’s highly aggressive nature (13). However, a subset of cases has demonstrated low ^18^F-FDG accumulation in metastatic nodules from choriocarcinoma, potentially influenced by hemorrhagic and/or necrotic components affecting ^18^F-FDG avidity (83). The reported SUVmax range for choriocarcinoma spans from 2.0 to 27.5, encompassing various studies (13, 16, 20, 21, 23, 26, 28, 30, 31, 33, 36, 45–47, 49, 51, 53, 54, 60–62, 64, 83).

The treatment approach for choriocarcinoma is contingent upon the disease’s stage (66). Non-gestational choriocarcinomas often receive a diagnosis in advanced or metastatic stages. Inaba et al. propose neoadjuvant chemotherapy to reduce tumor volume or high-dose chemotherapy before cytoreductive surgery (84). Currently, there is no standardized chemotherapy regimen for primary choriocarcinoma in males, with high-intensity chemotherapy regimens commonly employed, similar to those used for female choriocarcinoma. Frequently utilized chemotherapy protocols include EMA/CO (etoposide, methotrexate, actinomycin D, cyclophosphamide, and vincristine) and TP (paclitaxel and cisplatin). It is acknowledged that male patients often develop resistance to cytotoxic chemotherapy, leading to a grim prognosis. Factors such as poor response to chemotherapy, high disease burden, brain metastasis, and hemoptysis at the time of diagnosis correlate with shorter survival times in male primary choriocarcinoma patients, with a median overall survival of approximately 6 months and a 1-month mortality rate of 23.8% (85–90). In this study, the patient received treatment with etoposide, cisplatin, and bleomycin; however, metastases remained uncontrolled, resulting in the patient’s demise due to increased intracranial pressure and secondary epilepsy exacerbated by enlarged brain metastases. The overall survival was only 5 months. A consistent phenomenon observed in patients with poor prognoses was a rapid decrease in β-hCG to a lower level during treatment, followed by a sharp rise during disease relapse (91). Liu et al. suggest that, for advanced patients, a combination of adjuvant chemoradiotherapy with palliative surgery is recommended. If serum β-hCG drops to a normal level and residual lesions persist, salvage surgery to achieve an R0 status is considered worthwhile (76).

As the medical field advances, ongoing exploration and application of new treatment modalities persist. In a pre-clinical model, PD-L1 overexpression was identified in gestational trophoblastic specimens, suggesting the potential role of this ligand in tumor-immune evasion (92). Veras et al. (93) reported PD-L1 expression in human placentas and gestational trophoblastic diseases, including choriocarcinoma. Ghorani et al. (92) documented four cases of drug-resistant gestational trophoblastic neoplasia treated with pembrolizumab. Among these cases, all displayed PD-L1 overexpression, and three out of four patients achieved remission. The lack of response in one patient was attributed to the absence of tumor-infiltrating lymphocytes. In a recent phase II, single-arm, prospective trial (CAP 01), the efficacy and safety of camrelizumab (PD-1 inhibitor) combined with apatinib (vascular endothelial growth factor (VEGF) receptor inhibitor) were evaluated in patients with high-risk chemo-refractory or relapsed gestational trophoblastic neoplasia. The study included 20 patients (19 with gestational choriocarcinoma and one with placental site trophoblastic tumor). Notably, 50% of enrolled patients achieved a complete response with the combination of the two drugs, and none of the patients with a complete response experienced disease recurrence after discontinuation of the treatment (94). Contrastingly, in a study by Adra et al. (95), only one of three male patients with choriocarcinoma exhibited PD-L1 overexpression, and none of the three patients achieved an objective response to pembrolizumab treatment. These findings suggest that PD-1/PD-L1 blockade therapy may not be universally effective in all male patients. It is posited that the therapeutic efficacy of PD-1/PDL1 blockade varies based on clinicopathological features such as PD-L1 overexpression and the presence of tumor-infiltrating lymphocytes.

## Conclusion

In conclusion, non-gestational choriocarcinoma represents a rare entity in clinical practice and should be considered in young men presenting with gynaecomastia and elevated β-hCG levels alongside normal gonads. Thus, we advocate for a more comprehensive inquiry into medical history and a systematic examination. Male primary choriocarcinoma is often associated with widespread metastatic disease, rapid disease progression, and a poor prognosis. Early diagnosis is paramount for formulating an optimal management strategy and minimizing widespread metastasis to achieve the best clinical outcome. The case elucidated in this report contributes to a deeper understanding of the disease for clinicians. The ^18^F-FDG PET/CT examination not only visually delineates the lesion’s location and extent but also serves as a cornerstone for clinical tumor staging, providing valuable support for treatment monitoring and subsequent follow-up.

## Data availability statement

The original contributions presented in the study are included in the article/supplementary material, further inquiries can be directed to the corresponding authors.

## Ethics statement

Written informed consent was obtained from the individual(s) for the publication of any potentially identifiable images or data included in this article.

## Author contributions

WH: Conceptualization, Data curation, Writing – original draft, Writing – review & editing. ZZ: Conceptualization, Writing – review & editing. ZB: Formal analysis, Supervision, Writing – review & editing. XX: Conceptualization, Data curation, Writing – review & editing. LL: Data curation, Formal analysis, Writing – review & editing. ZS: Conceptualization, Data curation, Writing – review & editing. LK: Data curation, Formal analysis, Funding acquisition, Investigation, Supervision, Writing – original draft, Writing – review & editing.
